# Enhancing Facial Esthetics by Other Modalities

**DOI:** 10.1155/2011/513957

**Published:** 2011-08-14

**Authors:** Thotapalli Suman

**Affiliations:** Department of Prosthodontics, MNR Dental College, Sangareddy 502 294, India

## Abstract

Preprosthetic surgeries are generally dealt with surgical procedures performed to facilitate fabrication of prosthesis or improve the prognosis of prosthodontic care. In general the surgical procedures include various soft and hard tissue procedures which are restricted intraorally. Maxillofacial prosthodontics is not restricted to restorations performed intra-orally. Various extraoral surgical procedures have come into light in the recent past which helps to improve the prosthodontic outcome of craniofacial region. The current paper tries to elaborate various minimally invasive cosmetic reconstructive procedures and materials available in recent times.

## 1. Introduction

Historically, aesthetic facial surgery has been dominated by skin tightening and tissue removal procedures. These facelift procedures were described initially as the elevation, tightening, and excision of skin. Over the following decades, these procedures changed considerably which now include tightening of skin and deep structures, excision of excess tissue, composite or multiplane dissection, repositioning of ptotic tissue, and restoration of volume [1]. Tension restoration surgery continues to be useful but is most effective when combined with volume restoration. Today's patients do not want to miss work or play and desire “wash-and-wear” surgical procedures. Botulinum toxin type A (Botox), Restylane, nonablative lasers, and facial implants are examples of minimally invasive cosmetic facial surgery. 

## 2. Facial Implants

Implant materials for chin augmentation include Silicone, GORE-TEX, and porous polyethylene. Porous materials will make removal or revision of the implants difficult [2]. Postoperatively, if a nonporous material becomes a problem, it is easily repositioned or removed. Bone resorption is a result of pressure and muscle function. In patients with severely deficient chin and hypertrophic mentalis muscle, there may be resorption of underlying bone. Most patients with a mild-to-moderate deficient chin are well treated with an alloplastic implant [3]. 

### 2.1. GORE-TEX

**Figure 1:**
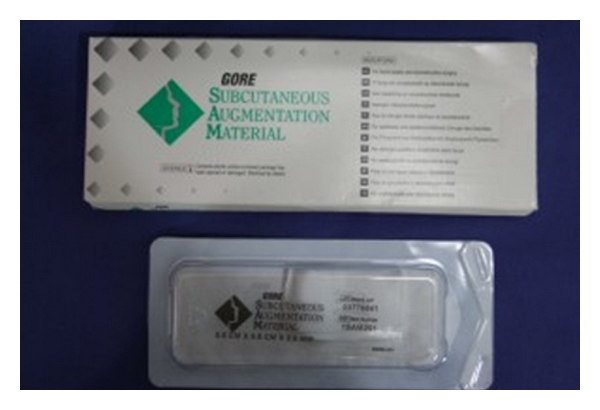
GORE-TEX.

GORE-TEX is a type of PTFE that has been used in humans as an implantable material for more than 26 years. It was first used in 1971 for human vascular grafts. In 1983, Neel used this polymer in rabbits and recommended its use in facial plastic surgery. Because vascular ingrowth is limited, this polymer has been shown to be only minimally integrated with the surrounding tissues. The pore sizes range from 0.5 sto 30 microns, which is not an ideal diameter for macrophage migration and tissue ingrowth but can be a favourable environment for bacterial invasion and infection (Figure 1).

Autogenous material, such as patient's own adipose tissue, can be harvested via syringe liposuction and injected into the desired area [4]. Other autologous materials include dermis, dermal-fat grafts, fascia, and galea. Human-derived donor materials for facial augmentation include AlloDerm, Cymetra (which is AlloDerm in a particulate micronized form), CosmoDerm and CosmoPlast, Fascian particulate fascia, and Fasciablast (which is fascia in different forms, including strands). In addition, xenografts, such as bovine-derived collagen fillers like Zyderm and Zyplast, are available. Dermalogen, a homologous compound composed of different forms of human collagen, is no longer available. More popular dermal fillers include different forms of hyaluronic acid such as Restylane, Perlane, Juvederm, and Hylaform. Synthetic materials include silicone (such as Silskin), polyalkylimide (such as Bio-Alcamid), hydroxyapatite (such as Radiesse), polylactic acid (such as Sculptra), and polymethylmethacrylate (such as ArteFill). All are useful for structural augmentation of the face.

### 2.2. PHDPE

Porous high-density polyethylene (PHDPE) is currently marketed in the United States under the trade name Medpor (Porex Surgical, Inc, College Park, Ga). It is formed by sintering small particles of high-density polyethylene to create a strong firm material that can be molded using hot water [5]. Pore sizes range from 100 to 250 *μ*m, with 50% being larger than 150 *μ*m. This is important because previous animal studies have shown that pore sizes greater than 100 *μ*m encourage tissue ingrowth [6, 7].

**Figure 2:**
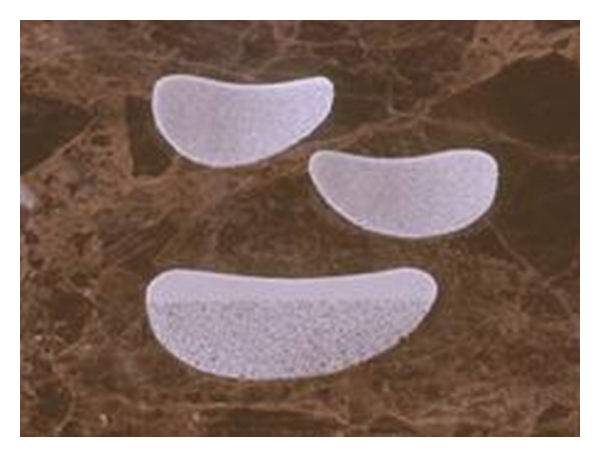
Porous high-density polyethylene (PHDPE).

Medpor comes in prefashioned models or can be tailored to specific patient's needs based on stereolithographic reconstruction from a 3-dimensional CT scan. Medpor is radiolucent on CT scans and MRI images, causing no interference with postoperative imaging, although a new version with titanium mesh embedded in the Medpor with minimal scatter that is MRI safe is radiopaque. The basic structure of Medpor is a simple carbon chain that makes it the reference standard for an inert substance in assays of tissue reaction [8]. Early studies of Medpor implants demonstrated fibroblast ingrowth that prevents capsule formation and promotes stabilization of the implant [9, 10]. De Potter and colleagues demonstrated fibrovascular ingrowth in vivo in patients who underwent orbital Medpor implantation (Figure 2).

They showed, through serial MRI examinations, enhancement as early as 1.5 months postoperatively. Over long periods, bone eventually incorporates at the implant-bone interface, providing additional stability [11].

### 2.3. Polytetrafluoroethylene

PTFE is a synthetic polymer that is carbon and fluorine based (CF2-CF2) and nonbiodegradable in the human body. It is biologically inert, adding to its appeal for use as implant material. Teflon is a type of PTFE in a paste form that has been used in the treatment of vocal cord disorders; however, its use has largely been abandoned because of injection difficulties and marked inflammatory reactions. Proplast [12], originally developed in 1970, was used to coat orthopedic joint implants. Later, Proplast II was used in facial plastic surgery. When used in temporomandibular joint (TMJ) surgery, Proplast, under the strong shearing forces caused by mastication, breaks apart and induces a significant inflammatory reaction. The Food and Drug Administration (FDA) ultimately recalled Proplast and Proplast II. 

### 2.4. Advanta Facial Implants (ePTFE)

**Figure 3:**
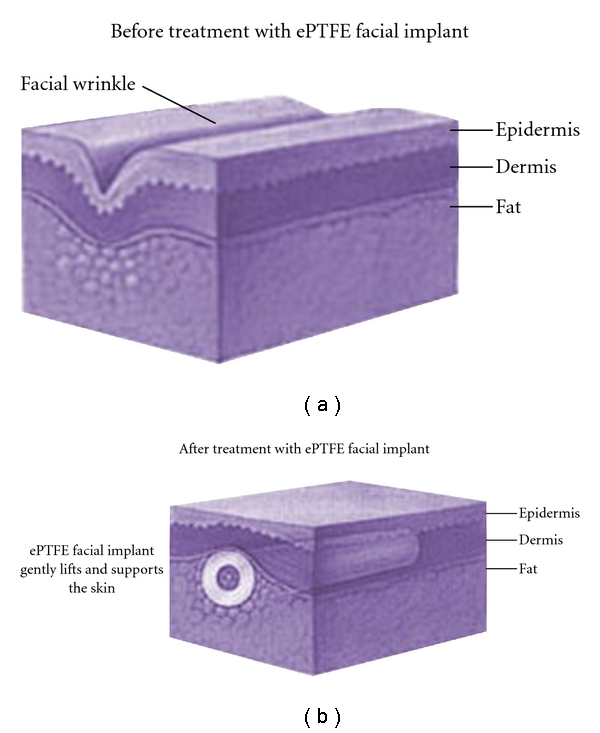
Expanded polytetrafluoroethylene (ePTFE).

Expanded polytetrafluoroethylene (ePTFE) is a woven polymer consisting of fibrils of PTFE that are connected via nodes of PTFE, creating a structure similar to mesh. ePTFE is FDA approved for facial augmentation. Originally, GORE-TEX strands used in the lip and nasolabial fold were not without problems. At times, the implants were palpable or extruded (Figure 3).

**Figure 4:**
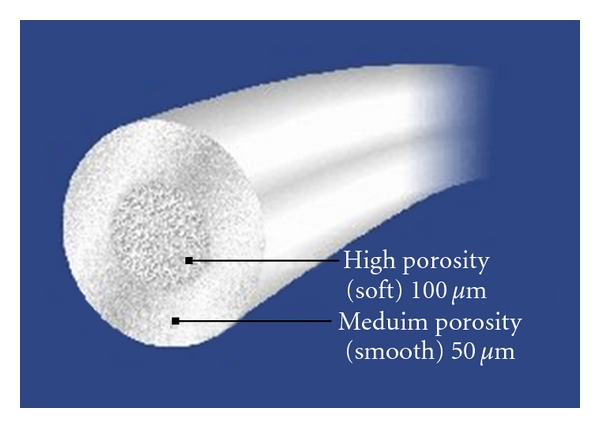
Advanta facial implants (ePTFE).

Facial implants have been used for many years and have included a plethora of materials. Contemporary choices for fillers include fat, expanded polytetrafluoroethylene ((ePTFE) GORE-TEX; W.L. Gore Co., Flagstaff, Arizona), Silicone, Silastic, polyethylene, bovine collagen, human collagen, hydroxyapatite, acrylic microspheres, lactic acid, dermis, fascia, and others [13]. Advanta facial implants (Atrium Medical, Hudson, New Hampshire) were different from the Gore-Tex implants used previously. They were more silky, soft, and pliable. The manufacturer attributes this look and feel to a sintering process in which the material is heated to impart these properties. In addition, the difference in this implant is the unique dual-core construction. Advanta implants are available in round or oval configurations and have an outer, medium-porosity smooth core of 50 microns and an inner, high- porosity soft core of 100 microns (Figure 4).

## 3. New Lip and Wrinkle Fillers

Over the millennia, various substances have been injected into the face, including wax, silicone, and animal products [14]. Contemporary cosmetic facial surgery includes many options to augment lips, folds, and wrinkles. For decades, bovine collagen has been the “gold standard” for facial filler augmentation in the United States [15]. An overview of injectable filler substances can be confusing. There exist many options, many fillers, many substances, many materials, and many claims of superiority. As stated earlier, bovine collagen (Zyplast, Zyderm; Inamed Corp., Santa Barbara, California) dominated the United States market for over 2 decades.

### 3.1. Zyplast

This FDA-approved product is collagen based and is composed of highly purified bovine dermal tissue. The constituent material is obtained from the hides of steer within a closed herd. The dermal filler ranges of products in this brand are fast, convenient, easy, nonsurgical tools to treat facial lines and wrinkles. Zyplast is mainly a cosmetic skin care treatment, injected into the skin to decrease the visibility of deep facial wrinkles, face folds and augment lip shape or size. It is a denser version of the common and popular injection Zyderm, both of which are made from collagen, a substance existing naturally within the skin tissue in the body. Conditions in which this product can be used include pronounced facial lines, scars, wrinkles, and lip border defining.

The treatment procedure involves four weeks prior testing which include administration of Zyplast test implant into the forearm to determine the sensitivity to the filler. Normally, only 3-4% of tested patients will have positive reaction. During treatment, the implant is injected into the skin by use of a fine gauge needle into the areas to be treated. This is a simple treatment, capable of being completed quickly and quite conveniently. The initial treatment generally lasts for 3–6 months, and repeated treatments can be undertaken to preserve the looks. Side effects include burning sensation, redness, bruising, stinging, and temporary swelling. The product was cleared for marketing by the Food and Drug Administration (FDA) in 1981 and 1985, respectively. Treatment results can always last for three to six months. Eventually, the injectable collagen breaks down, making it necessary for repeat treatments to maintain the desired results. In case treatments are discontinued, collagen will be reabsorbed, enabling the face to turn back to its original contours, naturally.

### 3.2. Zyderm

They were introduced in the early 1980s and until today remain widely used in over 40 countries with one million treatments having been undertaken. Due to the requirement of tests before treatment, its use has considerably reduced in the UK as a result of newly introduced products that do not require such tests. It is generically composed of Bovine dermal collagen plus 0.3% lignocaine and is made from cow skins.

Recommended tests before treatment should be performed four weeks prior to the treatment by administration of Collagen Test Implant, intradermally into the volar forearm. This is for the determination of possibility of the patient developing sensitivity to the implants. It is temporary in lasting since the collagen breaks down totally over time to a point where no trace of the filler can be found, hence repeat treatment is necessary. Product ranges include Zyderm 1 implant, Zyderm 2 implant, and Zyplast which is cross-linked with the chemical glutaraldehyde specifically for strengthening the collagen fibers. Effects will last for a period depending on lifestyle and implant placement. Expected effects are transient erythema (redness), pain, swelling, itching, implant area tenderness, and discoloration. In some cases, injected collagen can show on the skin as a small area, raised or white at the region of treatment. Credible tests for safety determination were carried out between 1976 and 1981 and the product was approved by the Food and Drug Administration (FDA).

### 3.3. Restylane

Restylane is FDA approved and is one of most popular fillers today. Restylane with natural hyaluronic acid makes treated areas fuller providing a natural, smooth, and attractive look. Restylane binds to water and replaces volume lost with sun damage and aging. It is used not only to fill in wrinkles, scars, and folds but also can provide a minifacelift by enhancing and filling in the cheeks hollows, under eye hollows, cheek bones, chin, and midface areas. The lips and earlobes are also wonderful areas for Restylane enhancement.

### 3.4. Artefill

Artefill is a permanent filler that is approved by the FDA. A skin test is necessary, and the product can be injected 1 month later. Artefill contains PMMA microspheres and purified bovine collagen gel with an anesthetic for comfort during injection. It is a dual-acting injectable wrinkle filler that provides immediate and long-lasting results. First Artefill visibly corrects the wrinkle, then the microspheres provide the permanent support the skin needs for enduring wrinkle correction. The collagen disappears and the beads promote the production of patients' own collagen around them and the result is permanent. It is an excellent filler for lines on the side of the mouth in someone who wants a permanent result.

## 4. Botox

Botulinum toxin is a protein produced by the bacterium *Clostridium botulinum* and is extremely neurotoxic. When introduced intravenously in monkeys, type A of the toxin exhibits an LD_50_ of 40–56 ng, type C1 around 32 ng, type D 3200 ng, and type E 88 ng, rendering the above types some of the most powerful neurotoxins known. Popularly known by one of its trade names, Botox or Dysport, it is used for various cosmetic and medical procedures.

Botox is a fully sequenced 1295 amino acid chain metalloprotease. It consists of a 97 kd heavy chain connected by a disulfide bond to a 52 kd light chain. The heavy chain binds to the cell membrane of the distal axon, which allows the light chain to penetrate the cell membrane into the cytoplasm. After it is inside the cell, it cleaves synaptosomal-associated protein 25, an essential protein in the mechanism that allows the acetylcholine-containing vesicles to fuse with the cell membrane, thus depositing the neurotransmitter into the synapse. By blocking this pathway, Botox prevents the presynaptic release of acetylcholine to the corresponding motor endplates, thereby directly affecting the nerve. Depending on the dose injected, Botox can produce a full range of effects, from mild weakness to full paralysis of a given muscle. At times, Botox is even used on selective parts of a single muscle. This exacting control is necessary to achieve facial rejuvenation while minimizing complications in the lower face. Clinically, the rejuvenating effects of Botox persist for approximately 3 months in the lower face after initial injection, but the duration of action typically increases after multiple treatments [16].

**Figure 5:**
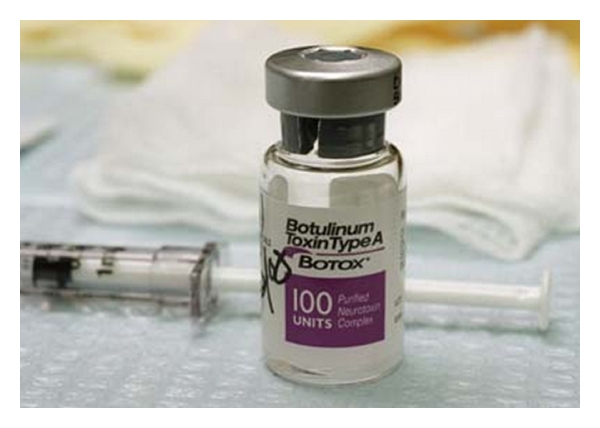
Botulinum toxin.

The first study to describe the therapeutic application of the toxin (in rhesus monkeys) was published by Scott (an ophthalmologist) and colleagues [17] in 1973. Scott's first publication that described its use in humans for strabismus appeared in 1980. The first study detailing the use of Botox for cosmetic reasons was published in 1992 [18]. Soon thereafter, other uses in the upper face and neck were described in the medical literature [19–21] (Figure 5). 

The pathogenesis of the wrinkle is important in determining how effective Botox will be in effacing it. The use of fillers in the lower face and the use of Botox for the upper face are stressed. When the rhytid is primarily caused by overhanging ptotic skin, surgery will probably be the most effective treatment. When the rhytid is primarily or at least significantly caused by muscular action deforming the overlying skin, Botox can be an extremely effective treatment in the lower face.

## 5. Commercial Names

BOTOX (onabotulinumtoxinA), RESTASIS (cyclosporine ophthalmic emulsion) 0.05%, LUMIGAN (bimatoprost ophthalmic solution) 0.03%, BOTOX Cosmetic (onabotulinumtoxinA), the JUVÉDERM family of dermal fillers, and the LAP-BAND Adjustable Gastric Banding System.

## 6. Conclusion

The materials and techniques described above are only few of the wide array of materials commercially available for facial reconstruction. The paper limits itself not to cover other procedures like fat augmentation, minimally invasive percutaneous collagen induction, suture suspension, and facial liposuction. The paper not only brings to light some of these recent trends but also has a purpose of considering these extraoral minimally invasive procedures as part of preprosthetic surgical preparation of the patient to provide with improved prosthodontic care.

## References

[1] Hamra ST (1998). The zygorbicular dissection in composite rhytidectomy: an ideal midface plane. *Plastic and Reconstructive Surgery*.

[2] Ramirez OM (1992). The subperiosteal rhytidectomy: the third-generation face-lift. *Annals of Plastic Surgery*.

[3] Zide BM, Pfeifer TM, Longaker MT (1999). Chin surgery: I. Augmentation-the allures and the
alerts. *Plastic and Reconstructive Surgery*.

[4] Sclafani AP, Romo T (1999). Alloplasts for nasal augmentation. *Facial Plastic Surgery Clinics of North America*.

[5] Lee S, Maronian N, Most SP (2005). Porous high-density polyethylene for orbital reconstruction. *Archives of Otolaryngology—Head and Neck Surgery*.

[6] Klawitter JJ, Bagwell JG, Weinstein AM, Sauer BW (1976). An evaluation of bone growth into
porous high density polyethylene. *Journal of Biomedical Materials Research*.

[7] Spector M, Flemming WR, Sauer BW (1975). Early tissue infiltrate in porous polyethylene implants
into bone: a scanning electron microscope study. *Journal of Biomedical Materials Research*.

[8] Rubin JP, Yaremchuk MJ (1997). Complications and toxicities of implantable biomaterials used in facial reconstructive and aesthetic surgery: a comprehensive review of the literature. *Plastic and Reconstructive Surgery*.

[9] Menderes A, Baytekin C, Topcu A, Yilmaz M, Barutcu A (2004). Craniofacial reconstruction with high-density porous polyethylene implants. *The Journal of craniofacial surgery*.

[10] Spector M, Harmon SL, Kreutner A (1979). Characteristics of tissue growth into Proplast and porous
polyethylene implants in bone. *Journal of Biomedical Materials Research*.

[11] Spector M, Flemming WR, Kreutner A, Sauer BW (1976). Bone growth into porous high density polyethylene. *Journal of Biomedical Materials Research*.

[12] Abramowicz S, Dolwick MF, Lewis SB, Dolce C (2008). Temporomandibular joint reconstruction after failed teflon-proplast implant: case report and literature review. *International Journal of Oral and Maxillofacial Surgery*.

[13] Brown LH, Frank PJ (2003). What’s new in fillers?. *Journal of Drugs in Dermatology*.

[14] Palkhivala A (2003). Injected silicone risks. *Dermatology Times*.

[15] Keefe J, Wauk L, Chu S, DeLustro F (1992). Clinical use of injectable bovine collagen: a decade of experience. *Clinical Materials*.

[16] Kane MAC Atrophy after repeated botox injections.

[17] Scott AB, Rosenbaum A, Collins CC (1973). Pharmacologic weakening of extraocular muscles. *Investigative Ophthalmology*.

[18] Carruthers JDA, Carruthers JA (1992). Treatment of glabellar frown lines with C. botulinum-a exotoxin. *Journal of Dermatologic Surgery and Oncology*.

[19] Garcia A, Fulton JE (1996). Cosmetic denervation of the muscles of facial expression with botulinum toxin: a dose-response study. *Dermatologic Surgery*.

[20] Carruthers A, Kiene K, Carruthers J (1996). Botulinum a exotoxin use in clinical dermatology. *Journal of the American Academy of Dermatology*.

[21] Kane MAC (1999). Nonsurgical treatment of platysmal bands with injection of botulinum toxin
A. *Plastic and Reconstructive Surgery*.

